# Chitosan-Templated Synthesis of Fe_2_O_3_, NiO, and NiFe_2_O_4_ Nanoparticles for Efficient Methylene Blue Dye Removal

**DOI:** 10.3390/polym17202750

**Published:** 2025-10-14

**Authors:** Amal Abdullah Alshehri, Laila Mohamad Alharbi, Maqsood Ahmad Malik

**Affiliations:** 1 Chemistry Department, Faculty of Science, King Abdulaziz University, P.O. Box 80203, Jeddah 21589, Saudi Arabia; aal.shehri@bu.edu.sa; 2 Department of Chemistry, Faculty of Science, Al-Baha University, P.O. Box 1988, Al-Baha 65799, Saudi Arabia; 3 Department of Chemistry, Faculty of Sciences, Jamia Millia Islamia, New Delhi 110025, India

**Keywords:** Ferrite, NiFe_2_O_4_ NPs, magnetic NPs, adsorption, MB dye, water remediation

## Abstract

Textile production contributes significantly to water pollution, making dye removal crucial for protecting water resources from toxic textile waste. The use of nano-adsorbents for water purification has emerged as a promising approach to removing pollutants from wastewater. Nickel Ferrite (NiFe_2_O_4_), Iron Oxide (Fe_2_O_3_), and Nickel Oxide (NiO) nanoparticles (NPs) were prepared via an auto-combustion sol–gel technique using chitosan as a capping and stabilizing agent. The prepared nanomaterials were characterized using various techniques such as XRD, UV-Vis DRS, FT-IR, Raman, EDX, SEM, and TEM to confirm their structure, particle size, morphology, functional groups on the surface, and optical properties. Subsequently, the adsorption of the methylene blue (MB) dye using the prepared nanomaterials was studied. NiFe_2_O_4_ NPs exhibited the best adsorption behavior compared to the mono-metal oxides. Moreover, all prepared nanomaterials were compatible with the pseudo-second-order model. Further investigations were conducted for NiFe_2_O_4_ NPs, showing that both the Freundlich and Langmuir isotherm models can explain the adsorption of the MB dye on the surface of NiFe_2_O_4_ NPs. Factors affecting MB dye adsorption were discussed, such as adsorbent dose, concentration of the MB dye, contact time, pH, and temperature. NiFe_2_O_4_ NPs exhibited a maximum removal efficiency of the MB dye, reaching 96.8% at pH 8. Different water sources were used to evaluate the ability of NiFe_2_O_4_ NPs to purify a wide range of water types.

## 1. Introduction

Globally, freshwater supplies are being threatened by increasing industrial pollution. Since water is a vital element for all living creatures, there is an escalating environmental risk associated with this [1]. The increasing use of dyes, especially reactive, direct, basic, and acidic dyes, which have high solubility in water and are difficult to remove from textiles using conventional methods, endangers human health and the environment [2,3,4,5]. Adsorption is the most economical, fast, and simple method for dye removal compared to other methods [1].

Methylene blue (MB) dye is a cationic dye from the phenothiazine family. This dye dissolves in water to give a blue solution. Moreover, it is widely used in various applications, such as biomedical applications, textiles, dyeing, food industries, and medicine. However, this dye poses significant dangers to human health because it is toxic, carcinogenic, and non-biodegradable. It is crucial to find a suitable method to remove this dye from wastewater [6].

In recent years, nanoparticles have been used in a wide variety of applications, including catalysis [7], medicine [8], batteries [9], sensors [10], fuel production [11], and cosmetics [12]. Among the different types of nanomaterials, ferrite nanoparticles (FNPs) with the general formula MFe_2_O_4_ (where M is a transition metal) have gained significant attention due to their characteristic properties [13]. Their high magnetic properties allow them to be easily separated from treated water, facilitating recovery and reuse. Consequently, nanoparticle residues in wastewater can be minimized, while the overall process cost is reduced [14,15]. Furthermore, they are characterized by large surface areas, small sizes, high reactivity, high blendability, and robust movement in solutions [16,17].

Nickel ferrite (NiFe_2_O_4_) has attracted considerable attention for its non-toxicity, low cost, high stability, and strong magnetic properties [18]. An inverse spinel cubic structure is predominantly found in nickel ferrite, with Fe^3+^ ions at tetrahedral sites and Ni^2+^ ions at octahedral sites. In response to changes in size and shape, it can exhibit paramagnetic, superparamagnetic, and ferromagnetic properties [19,20]. Additionally, it has a low band gap of 1.82 eV, indicating its capability to absorb visible light [21]. The properties of NiFe_2_O_4_ nanomaterials are significantly sensitive to the preparation methodology used in their synthesis. Various methods have been used for the preparation of NiFe_2_O_4_, including co-precipitation [22], Sol–Gel auto-combustion techniques [23], hydrothermal methods [24], microwave synthesis [25], and biosynthesis [21]. One of the most important issues in ferrite nanoparticle synthesis is to develop sustainable and efficient processes without agglomeration [26,27]. Biosynthesis, which uses plants, microorganisms, and biopolymers as capping, reducing, and stabilizing agents, has gained significant interest in recent years. It is characterized by being simple, cost-effective, and eco-friendly [28]. The Sol–Gel auto-combustion process is a straightforward one-pot procedure that is cost-effective, requires minimal time and external energy sources, and produces homogeneous crystals [29,30].

Chitosan is the second most abundant polysaccharide and is widely used as a capping agent in the preparation of metal oxide nanoparticles [31]. Moreover, it exhibits superior adsorption properties and selectivity towards metals and organic species due to its high content of hydroxyl and amino groups, which can easily be functionalized [32,33,34]. It is characterized by gel-forming capability, biodegradability, biocompatibility, and non-toxicity to living tissues, along with antibacterial, antifungal, and antitumor activity [35]. Nevertheless, it still has some limitations for use as a biosorbent, including low mechanical and chemical stability, difficult separation after adsorption, and solubility in acidic media [36]. In the preparation of metal oxide NPs, it can function as a capping, stabilizing, reducing, shape-directing, and size-controllable agent [31,37,38]. Therefore, ferrite nanoparticles that are mediated by chitosan display high adsorption capacities and can easily be separated from liquids after adsorption [39,40].

Sifontes et al. prepared highly porous CeO NPs with high surface area and small particle sizes by using chitosan as a template [41]. Additionally, Ben Amor et al. used chitosan as a capping agent in preparing ZnO NPs, which showed good dye degradability and antibacterial properties [42]. A chitosan-coated NiFe_2_O_4_ was prepared by Zeraatka et al. and demonstrated excellent removal of methyl orange and Congo red dyes [43]. Adeogun et al. suggested a one-pot co-precipitation method for chitosan/CoFe_2_O_4_ adsorbent, which was efficient for different types of anthraquinone dyes [44].

In the present study, chitosan-mediated nickel ferrite was prepared using a one-pot auto-combustion Sol–Gel technique, with chitosan as a gelling/capping agent and citric acid as a reducing agent. The as-prepared material was characterized using various microscopic and spectroscopic techniques. Nickel ferrite mediated by chitosan was investigated for its ability to remove methylene blue dye.

## 2. Experimental

### 2.1. Materials

Throughout these studies, all chemicals used were of high purity and did not require any additional purification. Furthermore, all solutions were prepared using deionized water. Chitosan (low molecular weight), citric acid powder (99.5% purity), acetic acid (99.8% purity), nickel(II) nitrate hexahydrate (>98% purity), iron(III) nitrate nonahydrate (≥98% purity), methylene blue (MB) (99.8% purity), sodium hydroxide (NaOH), and hydrochloric acid (HCl) were purchased from Sigma Aldrich, St. Louis, MO, USA.

### 2.2. Preparation of Fe_2_O_3_, NiO, NiFe_2_O_4_ NPs

**Figure 1:**
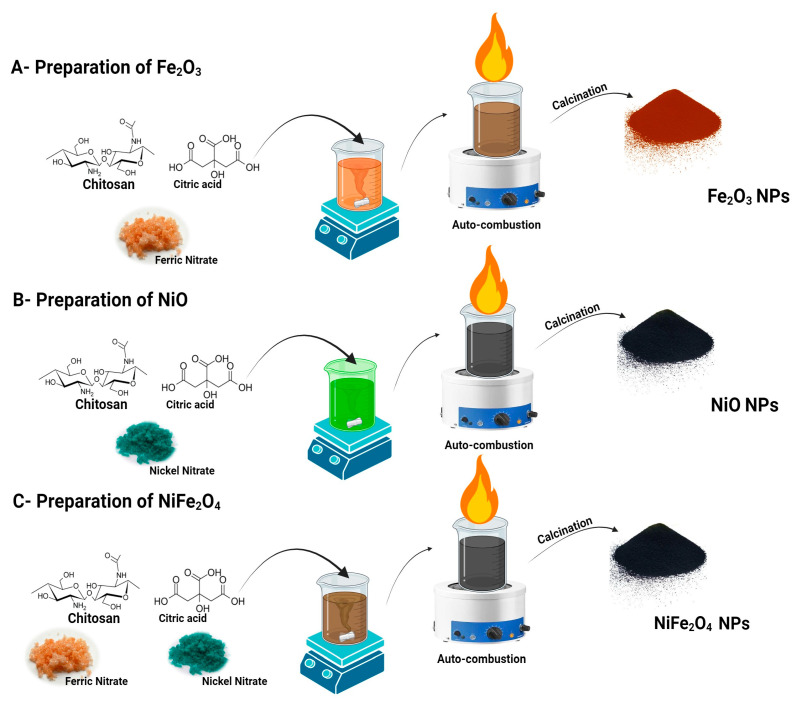
The schematic representation of the auto-combustion Sol–Gel synthesis of NiFe_2_O_4_ NPs using different chitosan as capping agent and citric acid as reducing agent.

Chitosan (3 g) was dissolved in a 0.1 M acetic acid solution. Ten grams of each metal precursor were dissolved in 50 mL of deionized water and added to the chitosan solution. The metal precursor solutions for NiFe_2_O_4_ were mixed before being combined with the chitosan. The solution was stirred continuously in a magnetic stirrer until homogeneous. Subsequently, 50 mL of 4% citric acid was added. At this point, the solution quickly changed to a deep black color, confirming the formation of nanoparticles (NPs). After two hours of magnetic stirring at 70 °C, a gel was formed. The gel was then auto-combusted and calcined at 500 °C for 4 h. Figure 1 shows the complete synthesis scheme of the prepared materials.

### 2.3. Characterization

#### 2.3.1. X-Ray Diffraction (XRD)

An X-ray diffractometer model k Alpha manufactured by (Thermofisher, Waltham, MA, USA) was used to examine the formation of NiFe_2_O_4_, Fe_2_O_3_, and NiO nanoparticles, as well as their crystallinity and particle size, using a Cu Kα X-ray source (1.54056 Å) at 40 kV, in the range of 5 to 80 degrees 2θ. Particle size was calculated using the Scherrer equation [45]:

(1) $$D=\frac{K\lambda }{\beta cos\theta }$$

where K = Scherrer constant, θ = Bragg angle, λ = wavelength of the X-ray beam used (1.54184 Å), and β = the Full width at half maximum (FWHM) of the peak.

#### 2.3.2. UV-Visible Spectroscopy

An X-ray diffractometer (Bruker’s D8 Advance) was used to examine the formation of NiFe_2_O_4_, Fe_2_O_3_, and NiO nanoparticles, as well as their crystallinity and particle size, using a Cu Kα X-ray source (1.54056 Å) at 40 kV, in the range of 5 to 80 degrees 2θ. Particle size was calculated using the Scherrer equation [45]:

αhν = A(hν − E_g_)_n_ (2)

where α = Absorption coefficient, A = Tailing parameter constant, h = Planck’s constant, E_g_ = Optical band gap, *ν* = Frequency of incident photons.

#### 2.3.3. Fourier Transform Infrared Spectroscopy (FTIR)

The functional groups of adsorbent sample play a critical role in improving adsorption. Therefore, a Fourier Transform Infrared Spectrometer (Model: Bruker Optics GmbH & Co., Rosenheim, Germany) was used to investigate the functional groups on the surface of the material.

#### 2.3.4. Transmission Electron Microscopy (TEM)

Transmission Electron Microscopes JET-1400FLASH (JEOL, Tokyo, Japan)was used to examine shape and size of the nanoparticles.

#### 2.3.5. Scanning Electron Microscopy with Energy Dispersive X-Ray Analysis (SEM-EDX)

(Hitachi S-3400N SEM, Hitachi, Chiyoda, Japan) was used to examine the morphology, purity, and elemental composition of the samples.

#### 2.3.6. Raman Spectroscopy

A Raman microscope (Bruker, Billerica, MA, USA) is used to examine the structural characteristics of nanomaterials.

### 2.4. Determination of MB Dye Concentration in Solution

**Figure 2:**
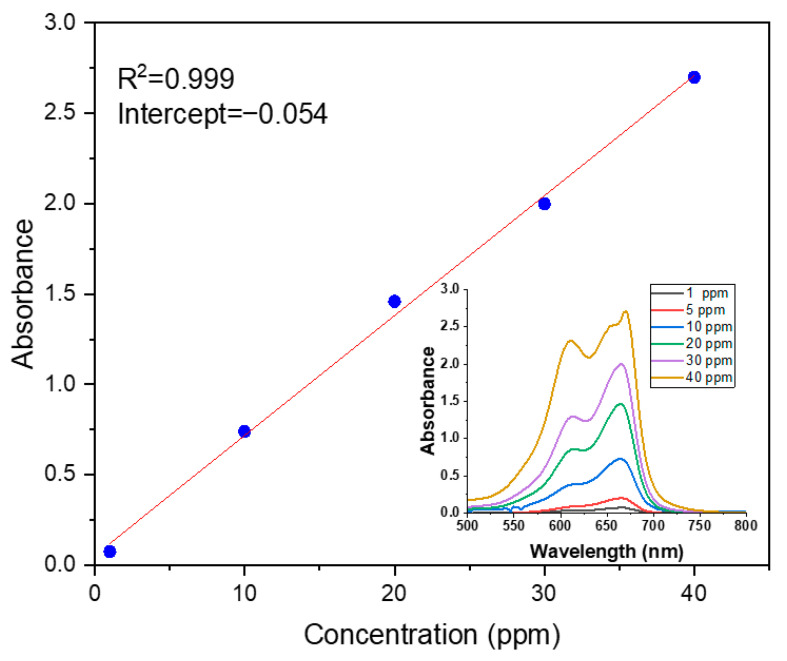
Calibration curve of UV-Vis absorption.

The concentration of MB dye was determined using a UV-VIS double-beam PC scanning spectrophotometer (UVD-2960) (Labomed, Inc., Los Angeles, CA, USA). A variety of concentrations of MB dye were prepared (1, 10, 20, 30, and 40 ppm). The standard curve of absorbance versus concentration is shown in Figure 2.

### 2.5. Adsorption Experiment

Adsorption experiments were conducted in a 50 mL capped conical flask using 25 mL of methylene blue (MB) dye solution under ambient conditions and normal pH, except when studying the effects of pH and temperature. The MB dye solution was filtered using a syringe filter (Type: Nylon 6, 0.22 μm, Tianjin Jinteng Experiment Equipment Co., Ltd., Tianjin, China). In this study, the impact of several factors, such as adsorbent dosage, MB dye concentration, pH, and temperature, on the adsorption and desorption of MB dye on the surface of as-prepared NiFe_2_O_4_ was examined. The removal percentage of MB dye and the amount of adsorbed MB dye per unit mass (mg/g) were calculated using the following equations [46]:

(3) $$\mathrm{Removal}\;\mathrm{of}\;\mathrm{MB}\;\mathrm{dye}\;(\%)\;=\;\frac{A_{o}-A_{t}}{A_{o}}\times 100$$

(4) $${q_{t}=C}_{o}-C_{t}\times \frac{V}{m}$$

where A_o_ and A_t_ are the absorbance of MB dye at t = 0 and t = t, C_o_ and C_t_ are the concentration (ppm) of MB dye at t = 0 and t = t, q_t_ is the amount of dyes adsorbed on the solid phase, V is the volume of solution in (L), m is the adsorbent weight in gram. q_t_ is the amount of dyes that adsorbed on the surface of solid phase (mg/g).

The kinetic adsorption experiments were conducted using 25 mL of MB dye solution (10 ppm) and 0.15 g of adsorbent under continuous magnetic stirring. The samples and the adsorbent were separated at varying times. A similar experiment, conducted at a different temperature, was performed to study the adsorption isotherm.

### 2.6. Water Samples Collection and Experiments

Tap water was collected from the laboratory at King Abdulaziz University in Jeddah. Before collecting the sample, the water was allowed to flow for seven minutes. Seawater samples were taken from the Red Sea. Both tap water and seawater were filtered twice to remove any solid particles. Mineral water was purchased from the Saudi market. Deionized and distilled water were obtained from the deionizer and distilled water units in the laboratory at King Abdulaziz University. Adsorption experiments were conducted in a 50 mL conical flask using 25 mL of methylene dye (10 ppm) solution and 0.15 g of adsorbent under ambient.

## 3. Results and Discussions

### 3.1. Characterization Results

#### 3.1.1. X-Ray Diffraction Results

**Figure 3:**
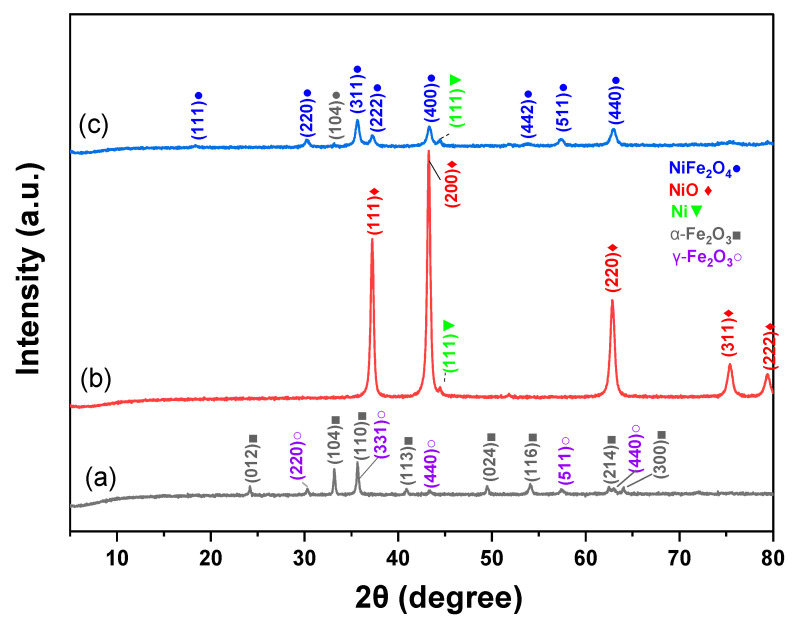
XRD patterns of (**a**) Fe_2_O_3_, (**b**) NiO, and (**c**) NiFe_2_O_4_ NPs.

The XRD patterns of Fe_2_O_3_, NiO, and NiFe_2_O_4_ NPS are shown in Figure 3. Fe_2_O_3_ patterns in Figure 3a were compared by JCPDS card file no. 33-0664 of α-Fe_2_O_3_ NPs and JCPDS card file no. JCPDS Card No. 019-0629 for Fe_3_O_4_ [47,48]. The XRD patterns exhibited distinguishable peaks at 2θ = 24.15°, 35.80°, 40.88°, 49.57°, 54.11°, 62.82°, and 64.16°, which correspond to the lattice planes (012), (104), (110), (113), (024), (116), (214), and (300) of α-Fe_2_O_3_ NPs, respectively [47].

The prominent diffraction peaks that appeared at 2θ = 30.31°, 35.66°, 43.29°, 57.46°, and 62.94° are related to (220), (331), (440), (511), and (440) Fe_3_O_4_ lattice planes, respectively [48,49]. According to JCPDS card file no. 78-0643, the diffraction peaks at around 2θ = 37.26°, 43.29°, 62.94°, 75.38°, and 79.53° shown in Figure 3b correspond to the (111), (200), (220), (311), and (222) lattice planes of NiO, respectively [50,51]. The peaks at around 33.18° and 44.48° are related to the iron oxide and metallic Ni NPs, respectively [52,53]. Based on JCPDS card file no. 10-0325, NiFe_2_O_4_ with cubic spinel structure is formed [54]. The peaks at around 2θ = 18.40°, 30.31°, 35.66°, 37.26°, 43.29°, 53.89°, 57.33°, 62.94°, and 75.27° correspond to the (111), (220), (311), (222), (400), (331), (511), (440), and (533) NiFe_2_O_4_ lattice planes of NiFe_2_O_4_, respectively, which agree with the results published in [52,55]. The crystallite sizes of Fe_2_O_3_, NiO, and NiFe_2_O_4_ NPs were 29.52, 19.09, and 13.00 nm, respectively.

#### 3.1.2. UV-Vis Spectra Analysis

**Figure 4:**
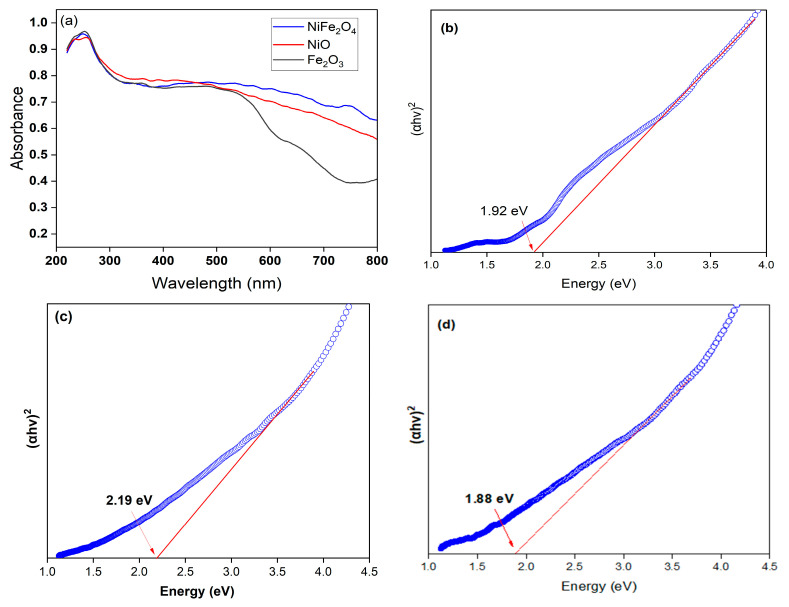
UV-visible diffuse reflectance spectra (UV-DRS) of (**a**) Fe_2_O_3_, NiO, NiFe_2_O_4_ NPs, (**b**) band gap of Fe_2_O_3_, (**c**) band gap of NiO, and (**d**) band gap of NiFe_2_O_4_.

Optical properties of Fe_2_O_3_, NiO, and NiFe_2_O_4_ samples have been investigated using UV-vis Diffuse reflectance spectra (DRS). Figure 4a shows the absorbance spectra of as-prepared samples. Fe_2_O_3_ shows a steep absorption edge in the range of 400–550 nm. This can be returned to the Fe-O electronic transition [56]. The pure NiO exhibits a 423 nm absorption band edge in the visible region. Interestingly, the optical absorbance spectrum of the NiFe_2_O_4_ has excellent absorption the visible region. The values of band gap energy (Eg) of Fe_2_O_3_, NiO, and NiFe_2_O_4_ in were found to be 1.92, 2.19, and 1.88 eV, respectively, as shown in Figure 4b–d.

#### 3.1.3. (FTIR) Analysis Spectra

**Figure 5:**
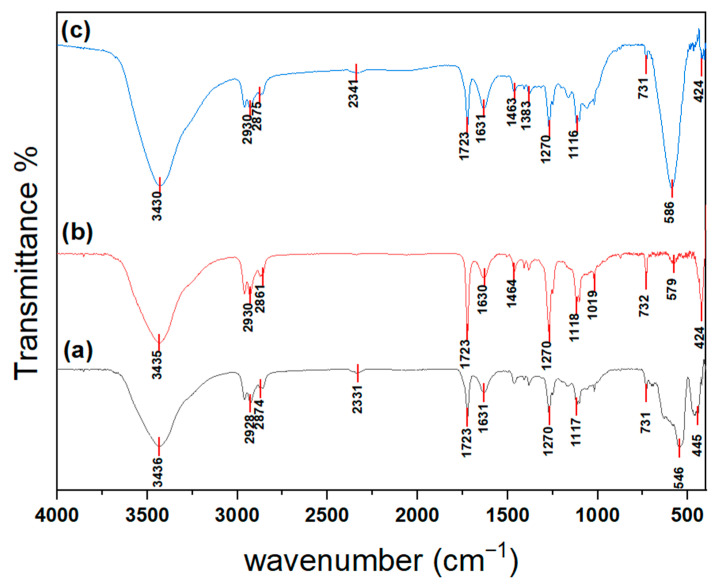
FTIR spectrum of (a) Fe_2_O_3_, (b) NiO, and (c) NiFe_2_O_4_ NPs.

FTIR spectra of Fe_2_O_3_, NiO, and NiFe_2_O_4_ nanoparticles (NPs) are shown in Figure 5 and were compared with the FTIR spectra of chitosan in [57]. The peaks at around 3430, 3435, and 3436 cm^−1^ may result from overlapping stretching vibrations of O-H and N-H bonds. The peaks at around 1630 and 1631 cm^−1^ may be related to the bending vibrations of O-H. The peaks at around 2861, 2874, and 2875 cm^−1^ are attributed to amide band I. Furthermore, the peaks at around 1462, 1463, and 1434 cm^−1^ are caused by vibrations of the O-H within the ring. Additionally, the peaks at 1379 and 1383 cm^−1^ are associated with C-H vibrations within the ring. The broad bands around 1116 cm^−1^ include stretching vibrations of glycosidic bonds, C–O, and C–O–C in the skeleton structure of chitosan [57,58,59].

In the FTIR spectrum of Fe_2_O_3_ (a), the two peaks at around 546 and 445 cm^−1^ correspond to the Fe–O stretching and bending vibrations of Fe_2_O_3_ [60]. Furthermore, the band at around 731 cm^−1^ is generated from the Fe-O-Fe bond stretching vibration [61]. Panel (b) shows the FTIR spectrum of NiO NPs; the bands at 579 and 424 cm^−1^ are assigned to Ni-O stretching vibration modes [62,63]. The FTIR spectrum of NiFe_2_O_4_ (c) shows two peaks at 586 and 424 cm^−1^, associated with stretching vibration bonds between nickel and oxygen ions at tetrahedral (Th) (Ni-O) sites and between iron and oxygen ions at octahedral (Oh) (Fe–O) sites. These observations indicate the formation of a spinel ferrite structure [64,65,66,67].

#### 3.1.4. Raman Analysis

**Figure 6:**
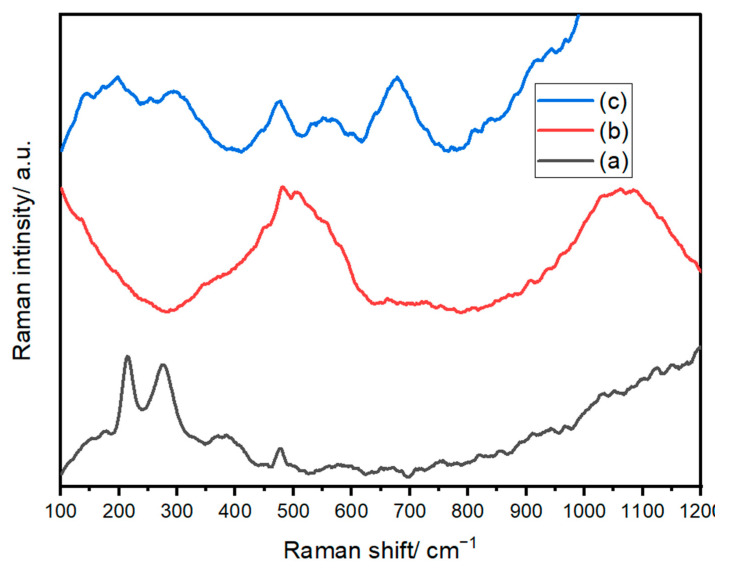
Raman spectra of (a) Fe_2_O_3_, (b) NiO, and (c) NiFe_2_O_4_ NPs.

Raman spectroscopy is used to confirm the formation of crystalline and amorphous structures and to assess the purity of the samples. The Raman spectra of Fe_2_O_3_ in panel (a) confirm the rhombohedral structure of Fe_2_O_3_ based on space group No. 148 R-3 [68]. The Raman spectra presented in Figure 6a depict the characteristic features of the freshly prepared Fe_2_O_3_ sample. This Raman spectrum prominently reveals significant peaks associated with α-Fe_2_O_3_. Simultaneously, discernible signals can be attributed to magnetite (Fe_3_O_4_) and maghemite (γ-Fe_2_O_3_).

Furthermore, some shifts in the spectral peaks are evident, which can be attributed to variations in the size and shape of the particles comprising the sample. Specifically, the strong peaks observed at 214, 275, 385, 475, and 576 cm^−1^ have been confidently attributed to the A_1_g and Eₑ modes of α-Fe_2_O_3_. Additionally, the presence of signals at 384 cm^−1^ and 650 cm^−1^ is likely linked to γ-Fe_2_O_3_ and Fe_3_O_4_, respectively, as indicated in reference [69].

In Figure 6b, the Raman spectrum of the prepared Ni/NiO nanosized structure reveals distinct characteristic peaks. Notably, the spectrum features prominent peaks at 500 and 1031 cm^−1^, corresponding to the first-order longitudinal (LO) mode and the second-order longitudinal (2LO) mode of NiO, respectively. These peaks indicate the vibrational characteristics of the Ni-O bonds within the structure. A sharp, well-defined emission around 557 cm^−1^ can be attributed to the presence of Ni defects [70], introducing a unique spectral signature. Additionally, the signals at 460 and 700 cm^−1^ correspond to the first-order transverse mode (TO) and the second-order transverse mode (2TO) [71], respectively, further contributing to the comprehensive characterization of the Ni/NiO nanosized structure. In Figure 6c, we observe the Raman spectrum of the prepared Ni/Fe_2_O_4_ compound, which exhibits the inverse spinel structure of AB_2_O_4_. These Raman spectra exhibit exceptional quality and closely resemble previously reported results for NiFe_2_O_4_. Particularly, we observe broad bands positioned at approximately 676, 551, 477, 294, and 197 cm^−1^. Each of these bands can be associated with specific vibrational modes, namely A_1_g(1), T_2_g(3), T_2_g(2), Eₑ, and T_2_g(1), as documented in reference [72]. It’s important to note that the broadening of the peaks in the spectrum of the as-prepared sample is a distinctive characteristic of its nanocrystalline structure. This feature underscores the unique structural attributes of the Ni/Fe_2_O_4_ compound, which are clearly reflected in its Raman spectrum.

#### 3.1.5. TEM Analysis

**Figure 7:**
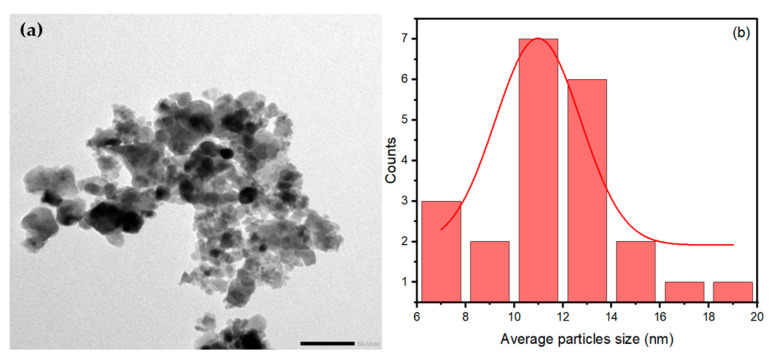
(**a**)TEM image of NiFe_2_O_4_ and (**b**) particle size distribution histogram.

The TEM image in Figure 7a shows the aggregated spherical morphology of NiFe_2_O_4_ nanoparticles (NPs). The average particle size of NiFe_2_O_4_ NPs is 11.50 nm, calculated from the TEM image using ImageJ software (https://imagej.net/ij/) based on 20 particles, as shown in Figure 7b. By comparing the average particle size of NiFe_2_O_4_ NPs obtained from the TEM image with the crystallite size obtained from the XRD patterns, it is evident that they are close in value. The aggregation of NiFe_2_O_4_ NPs can be attributed to the high magnetic properties of the NiFe_2_O_4_ NPs.

#### 3.1.6. SEM/EDX Analysis

**Figure 8:**
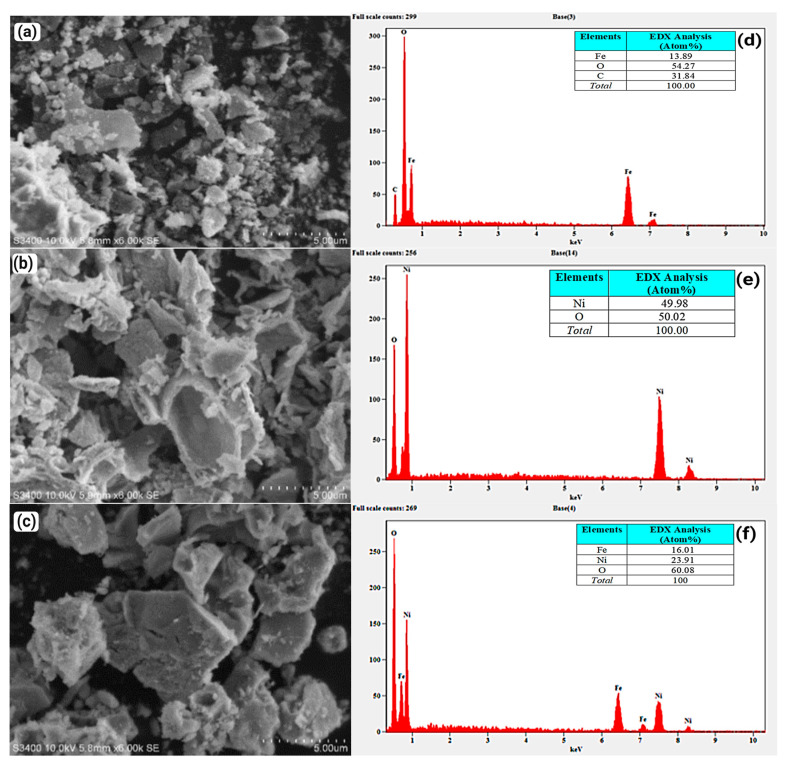
(**a**–**c**) are SEM images of Fe_2_O_3_, NiO, and NiFe_2_O_4_ respectively, (**d**–**f**) EDX spectra of Fe_2_O_3_, NiO, and NiFe_2_O_4_, respectively.

The SEM images of Fe_2_O_3_, NiO, and NiFe_2_O_4_ nanoparticles (NPs) are shown in Figure 8a–c. The particles are aggregated due to the magnetic properties of the NPs and the increased surface area, which leads to higher surface energies and strong interparticle attraction. This result agrees with [73]. The EDX spectra of the Fe_2_O_3_, NiO, and NiFe_2_O_4_ NPs are presented in Figure 8d–f. The EDX spectrum of Fe_2_O_3_ in Figure 8d confirms the formation of Fe_2_O_3_ with high purity. The carbon detected is a result of incomplete calcination of organic compounds [74]. In Figure 8e, the EDX spectrum of NiO shows the presence of Ni and O signals, confirming the high purity of the formed NiO NPs. The EDX spectrum of NiFe_2_O_4_ NPs in Figure 8f confirms the presence of Ni, Fe, and O in the nanocomposite structure. The high purity of the samples is confirmed by the absence of other peaks.

### 3.2. Removal Study of MB Dye

#### 3.2.1. Effect of NiFe_2_O_4_ Dosage

**Figure 9:**
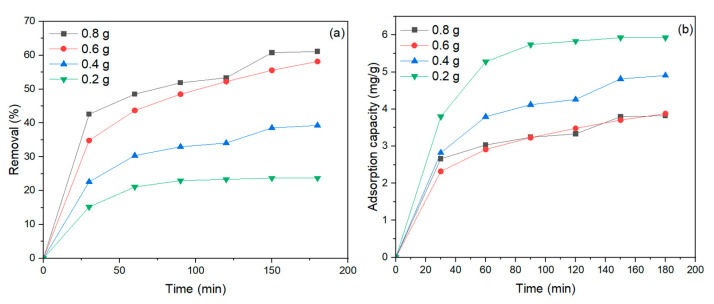
(**a**) Effect of NiFe_2_O_4_ dose on the removal (%) of MB dye vis contact time, (**b**) Effect of NiFe_2_O_4_ dose on equilibrium adsorption capacity and removal efficiency of MB dye.

To effectively remove MB dye, the amount of NiFe_2_O_4_ nanoparticles (NPs) used is an essential aspect. The adsorbent amount was adjusted from 0.2 to 0.8 g/100 mL, with a pH of 6, dye concentration of 40 mg/L, and mixing time of 180 min. As clearly shown in Figure 9a, as the amount of NiFe_2_O_4_ NPs increased, the MB dye removal also increased. This is consistent with the fact that when the amount of NiFe_2_O_4_ increases, the surface area also increases, leading to a greater number of active sites necessary for the adsorption process of MB dye [75]. Consequently, the adsorption capacity of MB dye decreased as the amount of NiFe_2_O_4_ increased, as shown in Figure 9b. This result agrees with [76,77], as higher adsorbent dosages tend to agglomerate, reducing adsorption efficiency. The percentage of MB dye removal by NiFe_2_O_4_ increased with longer contact times, as illustrated in Figure 9a. Initially, the removal of MB dye was rapid until a certain point, after which the dye settled on the adsorbent surface, and contact time did not significantly affect the rate of removal [78]. According to the experimental results, the removal of MB dye showed little change when increasing NiFe_2_O_4_ from 0.6 g to 0.8 g; therefore, 0.6 g of NiFe_2_O_4_ was used in the subsequent experiments.

#### 3.2.2. Effect of MB Dye Concentration

**Figure 10:**
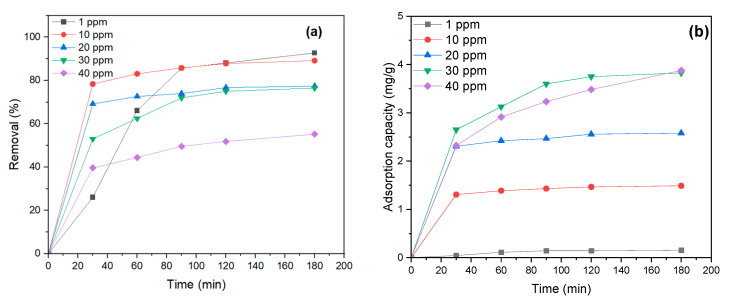
(**a**) Effect of MB dye concentration on the removal percentage and (**b**) effect of MB dye concentration on Adsorption capacity.

One of the essential factors in MB dye adsorption by NiFe_2_O_4_ is the initial concentration of MB dye. Different dye concentrations were tested to assess the influence of the initial MB dye concentration on the removal percentage (1–40 mg/L) with a 0.6 g dosage of NiFe_2_O_4_ and pH = 6. According to Figure 10a, MB dye removal decreases as the concentration increases, from 92.7% at 1 mg/L to 58.14% at 50 mg/L. This suggests saturation of binding sites on the NiFe_2_O_4_ surface as the MB dye concentration increases [79]. Moreover, the adsorption capacity decreases as the MB dye concentration rises. The removal of MB dye was rapid at first, then slowed down, reaching equilibrium after 120 min of contact time for initial concentrations from 10 to 50 mg/L, as shown in Figure 10b. Therefore, the contact time will be set to 120 min for the next experiment. There was no significant change in the removal of MB dye between 1 and 10 mg/L after 120 min, indicating that adsorption will be enhanced with a 10 mg/L initial MB concentration.

#### 3.2.3. Synergistic Effect

**Figure 11:**
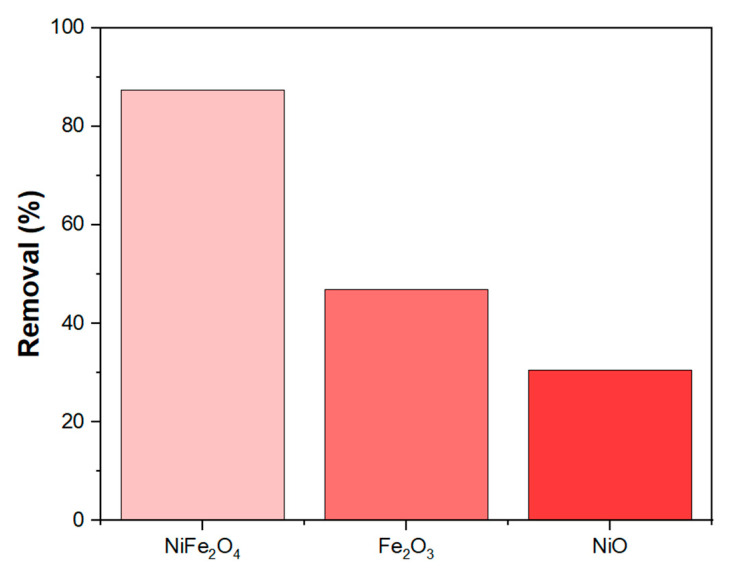
Exhibits synergistic effect in NiFe_2_O_4_ NPs.

The synergistic effect of NiFe_2_O_4_ nanoparticles (NPs) is clearly observed in Figure 11, where the MB removal efficiency of NiFe_2_O_4_ is significantly better compared to Fe_2_O_3_ and NiO under constant parameters. This result agrees with findings from [80,81], which suggest that the presence of two metals can provide active sites that work together synergistically to adsorb the MB dye.

#### 3.2.4. Kinetic Studies and Mechanism

Several kinetic models are commonly used to describe the adsorption process. Pseudo-first-order, Pseudo-second-order, Intra-particle diffusion, Liquid film diffusion, and Fractional power models were fitted to understand the nature of the adsorption of MB dye on the surface of NiFe_2_O_4_, Fe_2_O_3_, and NiO NPs.

The pseudo-first-order kinetic model (Lagergren equation) describes the correlation between the rate at which sorption sites on the adsorbents are occupied and the number of remaining unoccupied sites [82]. It can be expressed by the following linear equation:

(5) $$Ln(q_{e}-q_{t})=ln\;q_{e}-k_{1}t$$

A constant rate of adsorption is represented by k_1_ the pseudo-first order (min^−1^). The amount of MB dye adsorbed is q_e_, the amount of dye adsorbed at equilibrium, and q_t_, which is the amount of dye adsorbed at time t (min).

Pseudo-second-order kinetics model explains how the adsorption capacity of the adsorbent changes over time. This relationship can be determined using Equation (6):

(6) $$\frac{t}{q_{t}}=\frac{1}{k_{2}q_{e}^{2}}+\frac{t}{q_{e}}$$

where q_t_ is the equilibrium concentration of MB dye at equilibrium and q_e_ is the equilibrium concentration at time t (mins), and k_2_ is the pseudo-second-order rate constant (g/mg·min).

Elovich kinetic model describes second-order kinetics for a solid surface with heterogeneous energy, although it does not propose specific mechanisms for adsorption [83]. The linear equation of the Elovich model is expressed by Equation (7):

(7) $$q_{t}=\beta \ln(\alpha \beta )+\beta \ln t$$

where α represents the initial rate of adsorption (g/mg·min) and b indicates the extent of surface coverage and activation energy for chemisorption (mg/g·min).

The intra-particle diffusion kinetic model indicates that intraparticle diffusion limits the overall reaction rate when the catalyst particle size is large or when the intrinsic reaction rate is faster than the diffusion rate of reacting molecules within the catalyst pores. The degree of inhibition on the overall velocity depends on particle size, shape, intrinsic reaction velocity, and diffusion rate. Additionally, if intraparticle diffusion is the rate-limiting step, the amount adsorbed at any given time is directly proportional to the square root of the contact time and passes through the origin, as stated in the literature.

(8) $$q_{t}=K_{id}t^{1/2}+C\;$$

Liquid film diffusion model

(9) $$\ln\left(1-F\right)=-K_{fd}t$$

Fractional power model

(10) $$\ln q_{t}=\ln a+b\ln t$$

**Figure 12:**
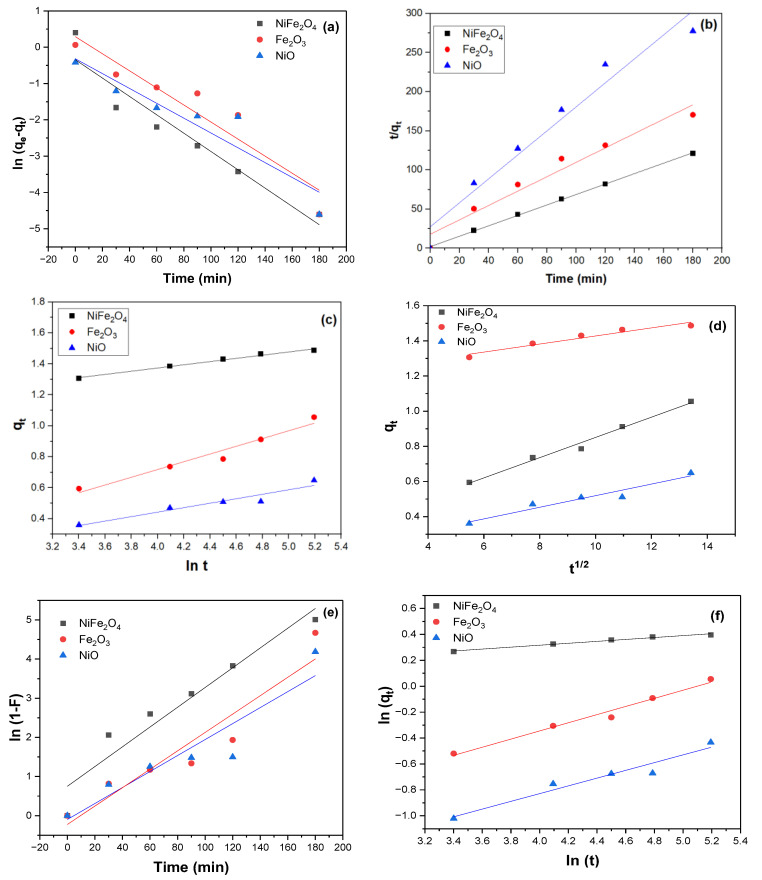
(**a**) Pseudo first order kinetics model, (**b**) Pseudo second order kinetics model, (**c**) Elovich kinetic model, (**d**) Intra-particle diffusion kinetic model. (**e**) Liquid film diffusion model, (**f**) Fractional power model for as prepared samples (experiment conditions: 0.6 g of adsorbent, 20 ppm of MB dye, pH = 6, and T = 291.5 K).

According to the pseudo-first-order plot of the experimental data Figure 12. The calculated equilibrium adsorption capacities (q_e_) are equal to 0.71, 1.33, and 0.73 mg/g for NiFe_2_O_4_, Fe_2_O_3_, and NiO NPs, respectively as shown in Table 1. These values do not match the experimental equilibrium adsorption capacities. Moreover, the R^2^ value is lower than that of the pseudo-second-order model. This indicates the pseudo-first-order model does not adequately describe the adsorption process. In contrast, the pseudo-second-order model showed a better fit to the experimental data, with the highest R^2^ value and a closer match between calculated (qcalc.) and experimental q(exp)values. This suggests that the adsorption of MB dye on the surface of NiFe_2_O_4_ or NiO can be described by the second-order rate equation. However, Fe_2_O_3_ NPs exhibited the highest R^2^value when analyzed using the intra-particle diffusion kinetic model, which can effectively describe its adsorption mechanism.

#### 3.2.5. Adsorption Isotherms

Adsorption isotherm studies are an integral part of understanding adsorption processes and determining which isotherm model best describes the adsorption behavior. In isothermal studies, the amount of MB dye adsorbed on the NiFe_2_O_4_ NPs was measured at different concentrations while keeping the temperature constant. This data was then used to fit Langmuir, Freundlich, and Temkin isotherm models to determine the best model that can describe the adsorption process of MB dye on the surface of NiFe_2_O_4_ NPs.

According to the Langmuir model isotherm theory, adsorbate molecules are monolayer homogeneously adsorbed on the solid surface because the active sites are the same energy [84,85]. The Langmuir linear equation is express as the following:

(11) $$\frac{1}{q_{e}}=\frac{1}{K_{L}q_{max}C_{e}}\frac{1}{C_{e}}+\frac{1}{q_{m}}$$

where C_e_ is the equilibrium concentration of the MB dye (mg/L), and q_e_ is the amount of MB dye adsorbed on NiFe_2_O_4_ NPs at equilibrium (mg/g). Moreover, K_L_ is the Langmuir constant (mg/g) that represents the monolayer adsorption capacity, and q_max_ is the coverage capacity of a monolayer at maximum.

According to the Freundlich isothermal model theory, adsorption takes place on a heterogeneous surface with multiple layers [86,87]. Freundlich’s linear equation is expressed as follows:

(12) $$lnq_{e}=lnK_{F}+\frac{1}{n}lnC_{e}$$

where C_e_ is the equilibrium concentration of the MB dye (mg/L), q_e_ is the amount of MB dye adsorbed on NiFe_2_O_4_ NPs at equilibrium, K_F_ is a constant that gives an approximate measure of adsorption capacity, and 1/n gives information about adsorption strength [88].

Temkin’s isotherm model theory assumes that all molecules in the layer have a linear decrease in the heat of adsorption with increasing surface coverage. This assumption implies that the interaction between the adsorbate molecules and the solid surface weakens as the surface becomes more crowded with adsorbed molecules [89]. The linear equation of Temkin isotherm can be expressed by the following equation:

(13) $$q_{e}=\frac{RT}{B_{T}}ln\;K_{T}+\frac{RT}{B_{T}}lnC_{e}$$

where $B_{T}\;$is the heat of sorption constant and K_T_ is Temkin isotherm constant [90].

**Figure 13:**
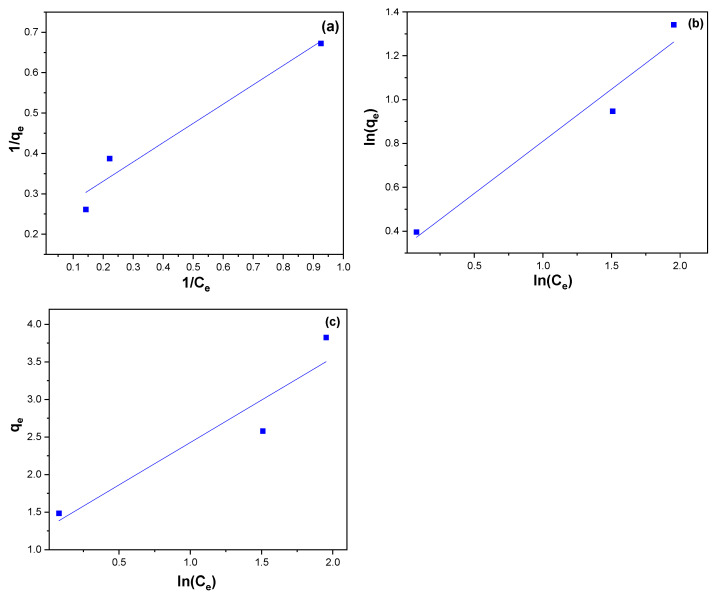
(**a**) Langmuir (**b**) Freundlich, and (**c**) Temkin isotherm linear equation for MB removal using NiFe_2_O_4_ NPs.

A linearized form of Langmuir equation for MB is illustrated in Figure 13a, the Freundlich linear equation is represented in Figure 13b, and Temkin isotherm linear equation is shown in Figure 13c. Langmuir, Freundlich, and Temkin regression coefficients are shown in Table 2. The correlation coefficient (R^2^) exhibited that the adsorption of MB on the surface of NiFe_2_O_4_ showed a better applicability with both Langmuir and Freundlich model. Although the Freundlich model fits the adsorption data just slightly better according to R^2^ value. Adsorbates are generally favorably adsorbed on adsorbents when n > 1. According to Table 2, NiFe_2_O_4_ is an appropriate dye for MB adsorption from aqueous solutions since n is greater than 1 [91]. The Langmuir isotherm (RL) value can be used to determine the favorability of an adsorption process. When the RL value is between 0 and 1 (0 < RL < 1), the adsorption process is considered favorable. If RL equals 1, the process is linear, while an RL value of 0 indicates irreversibility. When R_L_ exceeds 1 (R_L_ > 1), the process is deemed unfavorable. In our study, the RL values, a critical parameter of the Langmuir isotherm, fall between 0 and 1, signifying the favorable nature of the sorption process. On the other hand, because R^2^ is low, the Temkin model cannot reflect equilibrium isotherms for MB dye on the surface of NiFe_2_O_4_ NPs [92].

#### 3.2.6. Effect of pH

**Figure 14:**
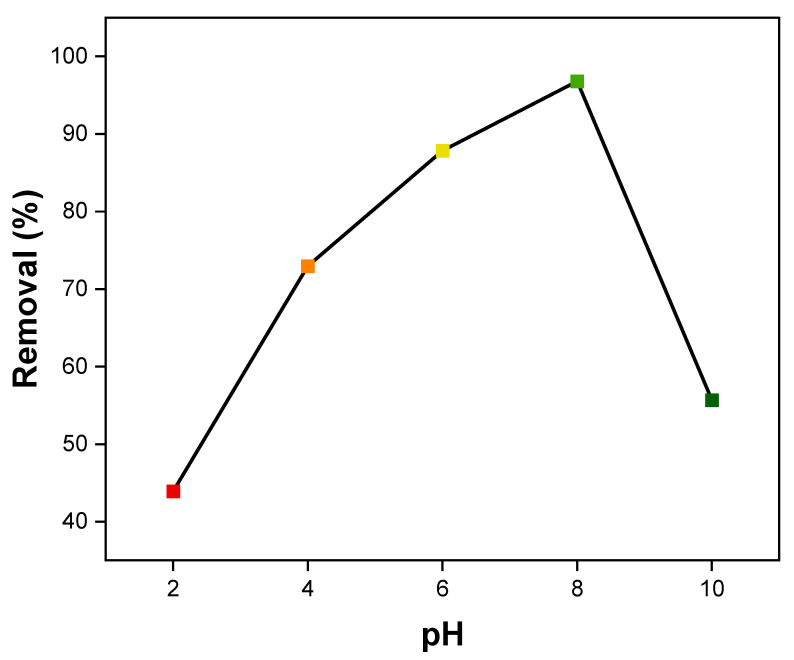
Effect of pH on MB dye adsorption by NiFe_2_O_4_ NPs.

There is a strong correlation between the adsorption of MB dye by NiFe_2_O_4_ nanocomposite and solution pH [93,94,95]. pH changes facilitate the ionization of adsorbate molecules and functional groups on adsorbent surfaces, thus affecting adsorption capacity. In general, surface charges on adsorbents change with pH in solution, impacting their adsorption rate [96]. The effect of pH on the MB dye removal percentage by NiFe_2_O_4_ was tested over a range from pH 2 to pH 10 using an initial MB dye concentration of 10 ppm, as shown in Figure 14. According to the graph, pH significantly affected the adsorption of MB on NiFe_2_O_4_. The amount of MB removed increased with the initial pH, reaching 96.8% at an initial pH of 8, before decreasing, which describes a bell-shaped curve. In high pH solutions, the adsorbent surfaces become more negatively charged, making them more likely to adsorb positively charged dyes. Conversely, the adsorption of negatively charged dyes is more efficient in low pH solutions, as the adsorbent surface becomes more positively charged [97]. Therefore, at low pH, the adsorption of the MB dye decreases, likely due to the positive charge on the NiFe_2_O_4_ NP surfaces caused by excess hydrogen ions, resulting in intermolecular repulsion with the positively charged MB dye. As pH increases, MB dye removal rises to a maximum at pH 8, attributed to the increased negative charge on the NiFe_2_O_4_ surface, which enhances electrostatic attraction with the MB dye. The adsorption of MB dye sharply decreases at pH 10, a behavior also reported in some studies [98,99,100]. This may be due to the hydrolysis of NiFe_2_O_4_ surfaces, which creates positively charged sites on the surface.

#### 3.2.7. Effect of Temperature and Thermodynamics

**Figure 15:**
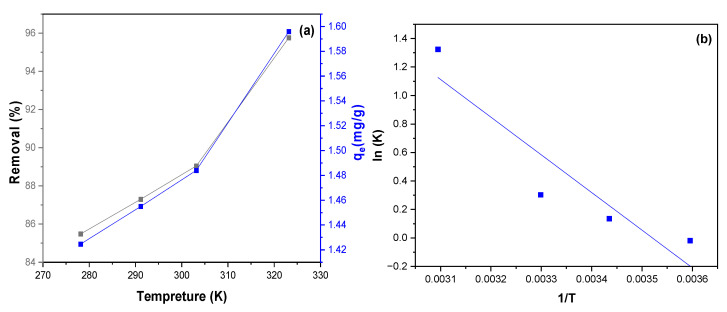
(**a**) the effect of temperature on the removal percentage of MB dye by NiFe_2_O_4_. (**b**) plot of lnD vis 1/T(K) to the thermodynamic parameter calculations for adsorption of MB dye by NiFe_2_O_4_.

The effect of temperature on the adsorption of MB dye by NiFe_2_O_4_ was investigated. As shown in Figure 15a,b, the relationship between the removal percentage and the adsorption capacity of MB adsorbed onto NiFe_2_O_4_ nanoparticles (NPs) at different temperatures (278.15, 291.15, 303.15, and 323.15 K) was examined under constant parameters. Increasing the temperature led to an increase in both the adsorption capacity and the removal percentage of MB dye, from 85.48% to 95.75%. The enhanced removal of MB dye by NiFe_2_O_4_ NPs with rising temperature can be attributed to several factors. Firstly, higher temperatures enhance the solubility of MB dye, leading to more collisions between the NiFe_2_O_4_ and the MB dye, thereby increasing the adsorption capacity. Furthermore, elevated temperatures can cause an expansion in the pore size of the NiFe_2_O_4_ surface, which subsequently increases its adsorption capacity. This expansion provides more available sites for MB dye molecules to attach, enhancing the overall adsorption process. Additionally, increasing temperature can improve the process by enhancing the mobility of MB dye molecules [101]. Thermodynamic studies were conducted to determine how temperature influences the adsorption of MB dye by NiFe_2_O_4_. As part of our investigation into the effect of temperature on adsorption, Equations (11)–(13) were used to determine the thermodynamic parameters relevant to the adsorption process:

(14) $$ln\;\left(D\right)=\frac{\Delta S}{R}-\frac{\Delta H}{RT}$$

(15) ΔG=ΔH−TΔS

(16) ΔG=−RTln(D)

where *D* is the constant of the adsorption equilibrium of the isotherm fits equal to ($\frac{q_{e}}{C_{e}}$), *R* is the universal gas constant (*R *= 8.3144 J/mol·K), and T is the temperature (K) [102].

According to Table 3, the results show that the enthalpy change (ΔH) is positive. This indicates that the adsorption process is endothermic, requiring energy input. As the temperature increases, more energy becomes available, leading to enhanced adsorption. The negative ΔG value indicates that the adsorption of MB dye onto the surface of NiFe_2_O_4_ nanoparticles (NPs) is thermodynamically favorable. This suggests that the process is spontaneous and can occur without additional energy input under standard conditions. The positive ΔS value indicates an increase in disorder or randomness between the MB dye solution and NiFe_2_O_4_ NPs during the adsorption process. Furthermore, the ΔG value ranges from 0 to −20 kJ/mol, and the ΔH value is less than 40 kJ/mol, indicating that the adsorption process is of a physical nature [103,104].

#### 3.2.8. Environmental Study

**Figure 16:**
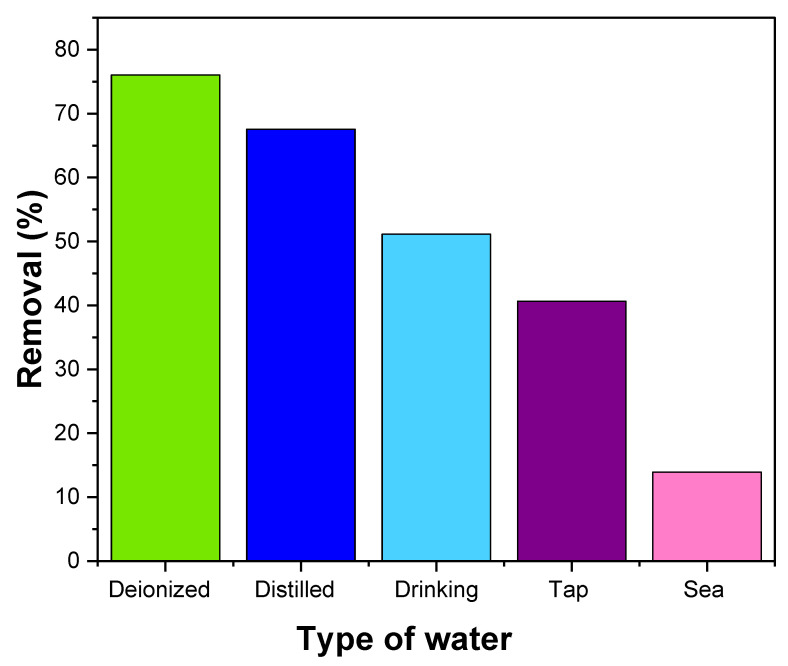
Effect of NiFe_2_O_4_ NPs in removal of MB dye from different types of water.

To investigate the suitability of NiFe_2_O_4_ for removing MB dye in actual environmental samples, various samples were collected from multiple sources, including saltwater from the Red Sea, distilled water from a laboratory distiller, drinking water purchased from the Saudi market, and tap water from the King Abdulaziz University laboratory. The MB dye removal percentages varied across the different samples, with deionized water showing the highest removal percentage, followed by distilled water, drinking water, tap water, and seawater, as depicted in Figure 16. The decrease in removal percentages can be attributed to the presence of pollutants in the samples. These pollutants may interact with the MB dye, competing for the active sites on the NiFe_2_O_4_ nanoparticles. As a result, the effectiveness of the dye removal process is reduced [105].

In Table 4, there is comparison between prepared NiFe_2_O_4_ NPs and the previous studied of metal ferrite NPs.

#### 3.2.9. Proposed Mechanisms of MB Dye on the Surface of NiFe_2_O_4_ NPs

**Figure 17:**
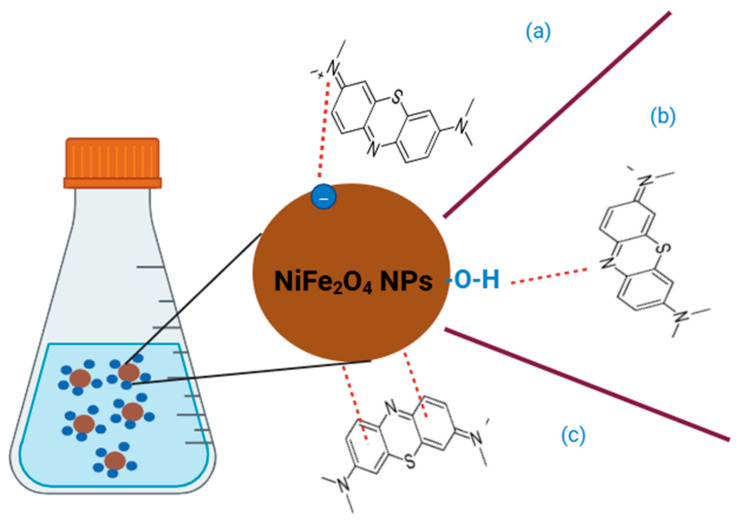
The mechanism of adsorption of MB dye on the surface of NiFe_2_O_4_ (**a**) Electrostatic forces, (**b**) Hydrogen bonds, and (**c**) π-π interactions.

As shown in Figure 17, the mechanism of adsorption of MB dye on the surface of NiFe_2_O_4_ involves: (1) Electrostatic forces, (2) Hydrogen bonds, and (3) π-π interactions. Electrostatic forces, the cationic MB dye is attracted to the negative surface of NiFe_2_O_4_ NPs. Hydrogen bonds, it arises due to attraction between MB dye and functional group on the surfaces of NiFe_2_O_4_ NPs. π-π interactions, it produces from the interaction between aromatic rings in MB dye and the surface of NiFe_2_O_4_ NPs [113].

## 4. Conclusions

In this study, NiFe_2_O_4_, Fe_2_O_3_, and NiO NPs were successfully prepared using a simple green method. The characterization results confirm the structure of as-prepared materials with the presence of metallic Ni NPs that grew during the preparation step on the surface of NiO and NiFe_2_O_4_ NPs. The NiFe_2_O_4_ NPs showed the best optical properties when compared with as-prepared mono-metal oxides. FTIR confirms the presence of functional groups that can enhance the adsorption process. NiFe_2_O_4_ showed the best MB dye removal performance compared to NiO and Fe_2_O_3_ NPs. Moreover, the adsorption process of NiFe_2_O_4_, NiO and Fe_2_O_3_ NPs follows the pseudo-second-order kinetic model with K_2_ = 0.218 (g/mg·min). Freundlich isothermal model theory gave the best applicability to describe the adsorption of MB on the surface of NiFe_2_O_4_ NPs. Moreover, thermodynamic studies showed that the adsorption of the MB dye on the surface of NiFe_2_O_4_ is spontaneous, endothermic, and feasible in nature. Furthermore, NiFe_2_O_4_ NPs showed good performance in removing the MB dye from different types of water. This indicates that the prepared NiFe_2_O_4_ NPs are considered a promising material for future water purification applications.

## Figures and Tables

**Table 1:** Different Kinetic models parameters for MB dye adsorbed NiFe_2_O_4_, Fe_2_O_3_, and NiO NPs.

**Pseudo first order kinetics model**				
	**q_e(exp.)_ (mg/g)**	**q_e(calc.)_ (mg/g)**	***K*_1_ (min^−1^)**	**R^2^**
**NiFe_2_O_4_**	1.45	0.71	0.025	0.926
**Fe_2_O_3_**	1.06	1.33	0.024	0.895
**NiO**	0.65	0.73	0.02	0.873
**Pseudo second order kinetics model**				
	**q_e(exp.)_ (mg/g)**	**q_e(calc.)_ (mg/g)**	***k*_2_ (g/mg**·**min)**	**R^2^**
**NiFe_2_O_4_**	1.45	1.5	0.218	0.999
**Fe_2_O_3_**	1.06	1.09	0.047	0.957
**NiO**	0.65	0.65	0.086	0.956
**Elovich kinetic model**				
	**α (g/mg**·**min)**		**β (mg/g**·**min)**	**R^2^**
**NiFe_2_O_4_**	10^5^ × 1.00		0.1	0.988
**Fe_2_O_3_**	1.29		0.25	0.955
**NiO**	2.72		0.14	0.919
**Intra-particle diffusion kinetic model**				
	**k_d_ (mg/g,min^1/2^)**		**C (mg/g)**	**R^2^**
**NiFe_2_O_4_**	0.02		1.2	0.941
**Fe_2_O_3_**	0.06		0.27	0.987
**NiO**	0.03		0.19	0.938
**Liquid film diffusion model**				
	***K_fd_* (min^−1^)**		**R^2^**	
**NiFe_2_O_4_**	−0.75		0.926	
**Fe_2_O_3_**	0.22		0.896	
**NiO**	0.098		0.873	
**Fractional power model**				
	**A**	**b**	**ab**	**R^2^**
**NiFe_2_O_4_**	1.02	0.07	0.08	0.985
**Fe_2_O_3_**	0.2	0.31	0.06	0.98
**NiO**	0.13	0.3	0.04	0.944

**Table 2:** Langmuir, Freundlich, Temkin Adsorption Isotherm.

**Langmuir Adsorption Isotherm**		
**K_L_ (L/mg)**	**q_(max)_ (mg/g)**	**R^2^**
0.49	4.25	0.978
**Freundlich adsorption isotherm**		
**K_F_**	**1/n**	**R^2^**
1.4	0.47	0.98
**Temkin adsorption isotherm**		
**K_T_(L/mg)**	**B_T_ (J/mol)**	**R^2^**
3.16	1.13	0.945

**Table 3:** Thermodynamic data for the adsorption of MB dye by NiFe_2_O_4_.

Thermodynamic Parameter	Calculated Value
ΔH (kJ/mol)	21.97
ΔS ( J.mol^−1^ K^−1^)	77.34
ΔG (kJ/mol)	−0.54

**Table 4:** Comparison of the adsorption of organic dyes onto different metal ferrite adsorbents.

Adsorbent	Dye	Adsorption Capacity or %	Ref.
CoFe_2_O_4_	CR dye	190.5 mg/g	[106]
ZnF_2_O_4_ @ Silica	MB dye	94.4%	[107]
NiFe_2_O_4_ NPs	MB dye	72 mg/g	[108]
Activated carbon/ZnFe_2_O_4_	AC dye	208.29 mg/g	[109]
ZnFe_2_O_4_ NPs	EB dye	46 mg/g	[110]
NiFe_2_O_4_ Nanofibers	CR dye	~97%	[111]
EG@NiFe_2_O_4_	CR dye	88.56%	[112]
NiFe_2_O_4_ NPs	MB dye	96.8%	This study

## Data Availability

The original contributions presented in this study are included in the article. Further inquiries can be directed to the corresponding author.
